# Interferon lambda inhibits dengue virus replication in epithelial cells

**DOI:** 10.1186/s12985-015-0383-4

**Published:** 2015-09-28

**Authors:** Helen K. Palma-Ocampo, Juan C. Flores-Alonso, Verónica Vallejo-Ruiz, Julio Reyes-Leyva, Lilian Flores-Mendoza, Irma Herrera-Camacho, Nora H. Rosas-Murrieta, Gerardo Santos-López

**Affiliations:** Laboratorio de Biología Molecular y Virología, Centro de Investigación Biomédica de Oriente, Instituto Mexicano del Seguro Social, Metepec, Puebla México; Laboratorio de Inmunología, Centro de Investigación Biomédica de Oriente, Instituto Mexicano del Seguro Social, Puebla, México; Posgrado en Ciencias Químicas, Benemérita Universidad Autónoma de Puebla, Puebla, México; Laboratorio de Bioquímica, Centro de Química, Instituto de Ciencias, Benemérita Universidad Autónoma de Puebla, Puebla, México

**Keywords:** Dengue, DENV, DENV-2, Interferon λ, SOCS, IL-28A, IL-28B, IL-29, IFN-III, IFN-λ

## Abstract

**Background:**

In viral disease, infection is controlled at the cellular level by type I interferon (IFN-I), but dengue virus (DENV) has the ability to inhibit this response. Type III interferon, also known as lambda IFN (IFN-III or IFN-λ), is a complementary pathway to the antiviral response by IFN-I. This work analyzed the IFN-λ (IFN-III) mediated antiviral response against DENV serotype 2 (DENV-2) infection.

**Methods:**

Dengue fever patients were sampled to determine their IFN-λ levels by ELISA. To study the IFN-λ response during DENV infection we selected the epithelial cell line C33-A, and we demonstrated that it is permissive to DENV-2 infection. The effect of IFN-λ on virus replication was determined in these cells, in parallel to the expression of IFN-stimulated genes (ISGs), and Suppressor of Cytokine Signaling (SOCS), genes measured by RT-qPCR.

**Results:**

We found increased (~1.8 times) serological IFN-λ in dengue fever patients compared to healthy blood donors. IFN-λ inhibited DENV-2 replication in a dose-dependent manner *in vitro*. The reduction of viral titer corresponded with increased ISG mRNA levels (MX1 and OAS1), with the highest inhibition occurring at ISG’s peak expression. Presence of IFN-negative regulators, SOCS1 and SOCS3, during DENV-2 infection was associated with reduced IFN-λ1 expression.

**Conclusions:**

Evidence described here suggests that IFN-λ is a good candidate inhibitor of viral replication in dengue infection. Mechanisms for the cellular and organismal interplay between DENV and IFN- λ need to be further studied as they could provide insights into strategies to treat this disease. Furthermore, we report a novel epithelial model to study dengue infection *in vitro*.

## Background

Dengue is an important public health problem: Using updated mapping approaches, there are 390 million estimated dengue infections per year worldwide, of which 96 million manifest clinically at some level of severity [1]. This leads to half a million hospitalizations and 25,000 deaths, mainly in children [2]. The etiological agent of dengue disease is the dengue virus (DENV), which is responsible for dengue fever (DF), dengue hemorrhagic fever (DHF) and dengue shock syndrome (DSS).

*Dengue virus*, a member of the *Flaviviridae* family, is an enveloped virus containing a ~11 kb genome of positive single-stranded RNA which encodes three structural proteins (C, pr-M, E) and seven nonstructural proteins (NS1, NS2A, NS2B, NS3, NS4A, NS4B, NS5) [3]. Four serotypes of dengue virus (DENV-1, DENV-2, DENV-3, and DENV-4) cause DF and more severe manifestations like DHF and DSS [4].

Dengue virus is transmitted by female mosquitoes mainly of the species *Aedes aegypti* and *A. albopictus*. The disease is widespread throughout the tropics, with local spatial risk variations influenced strongly by rainfall, temperature and unplanned rapid urbanization [5].

In many viral diseases, infection is controlled at the cellular level by type I interferon (IFN-α/β) produced in response of viral RNA. Recognition of IFN-I by its specific cell receptors activates the JAK-STAT signaling pathway, which leads to the establishment of an antiviral state in the cell. However, viruses possess diverse strategies to circumvent IFN effects [6].

DENV, like other viruses, has the ability to inhibit this innate response, which may contribute to its pathogenesis. DENV virus induces the activation and release of high levels of chemokines and pro-inflammatory cytokines in human dendritic cells, although it is a weak inducer of IFN-I, as it blocks IRF-3 phosphorylation, impairing subsequent IFN-α/β production [7].

There are three types of IFN. Type I comprises several subtypes of IFN-α and several other isoforms such as IFN-β,-ε,-κ,-δ,-ω,-τ and -ζ [8, 9]. Type II contains only one member, IFN-γ. The latest identified group, type III, comprises IFN-λ1, IFN-λ2, IFN-λ3 (also named interleukin [IL]-29, IL-28A and IL-28B, respectively) and IFN-λ4 [10–12], but there is limited information about their expression and biological functions. Furthermore, the role of IFN-III during DENV infection is unexplored.

IFN-λ specific signaling occurs through its receptor on the cell membrane, which consists of two subunits: IL28R1 (also named IFN-λR1, IL28Rα) and IL-10 receptor 2 (IL-10R2) [8, 10, 11]. Most cells express the IL-10R2 subunit, but IL28R1 distribution appears to be much more restricted. The receptor for IFN-λ is expressed preferentially in cells of epithelial origin and in some cells of the immune system [8, 10, 13].

Binding of IFN-λ to its receptor leads to the activation of JAK1 and Tyk2, which mediate the phosphorylation of STAT1 and STAT2 proteins. STAT phosphorylation leads to their dimerization, binding to IRF9 and translocation into the nucleus where they regulate the expression of specific genes. IFN-III has similar functions to IFN-I, inducing an antiviral state in cells [8, 10, 13] and mediates the antiviral response against several human pathogens including hepatitis B virus, hepatitis C virus and human immunodeficiency virus [13–15]. The restricted expression of its receptor suggests that the IFN-λ could exert an antiviral role on certain cell types [16, 17].

There is no commercial vaccine or specific antiviral treatment for dengue; thus, the development of efficient therapeutic strategies against this common and sometimes severe infection is urgent. The antiviral activity of interferons has been observed in diverse clinical studies showing a relative efficiency against different viral infections. In 1984, Limonta Vidal et al. [18] introduced the use of leukocyte interferon to treat children with dengue, showing a significant decrease in the number of complications and deaths when interferon was applied. However, the clinical use of interferon to treat dengue is not common, possibly due to IFN-α side effects associated with its systemic activity. IFN- λ is an attractive treatment alternative because it elicits a satisfactory antiviral activity with only a minor secondary response [19, 20]. Nevertheless, DENV has developed mechanisms to evade the immune response. Therefore, the precise analysis of IFN- λ response during dengue infection is crucial to evaluate/predict its potential clinical uses in this disease.

The present work focuses on the effect in epithelial cells of IFN-λ (IFN-III) on the DENV-2 infection. We found higher levels of IFN-λ in dengue fever patients compared with healthy blood donors and we provide evidence that endogenous and exogenous IFN-λ inhibit DENV-2 replication in the C33-A cell line.

## Results

### IFN-λ levels in patients with dengue fever

**Fig. 1:**
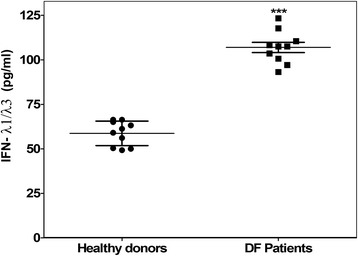
Dengue fever patients enhance their IFN-λ1 levels in blood. IFN-λ1/λ3 levels (pg/ml) in serums from clinical dengue patients and healthy donors were measured by ELISA. The experiment was done by triplicate and reproduced once. Error bars represent standard deviations, *** means *p* < 0.001

To detect the presence of IFN-λ during DENV infection, serum samples from patients with dengue fever were analyzed by ELISA. Levels of IFN-λ (λ1/λ3) were determined in serum samples from 10 patients with dengue fever and 10 healthy blood donors. Figure 1 shows the value distribution for each group; the mean was 106.9 ± 9.05 pg/ml for DF patients and 58.71 ± 6.86 pg/ml (*p* < 0.001) for healthy donors.

### C33-A cells are permissive to DENV-2 infection

**Fig. 2:**
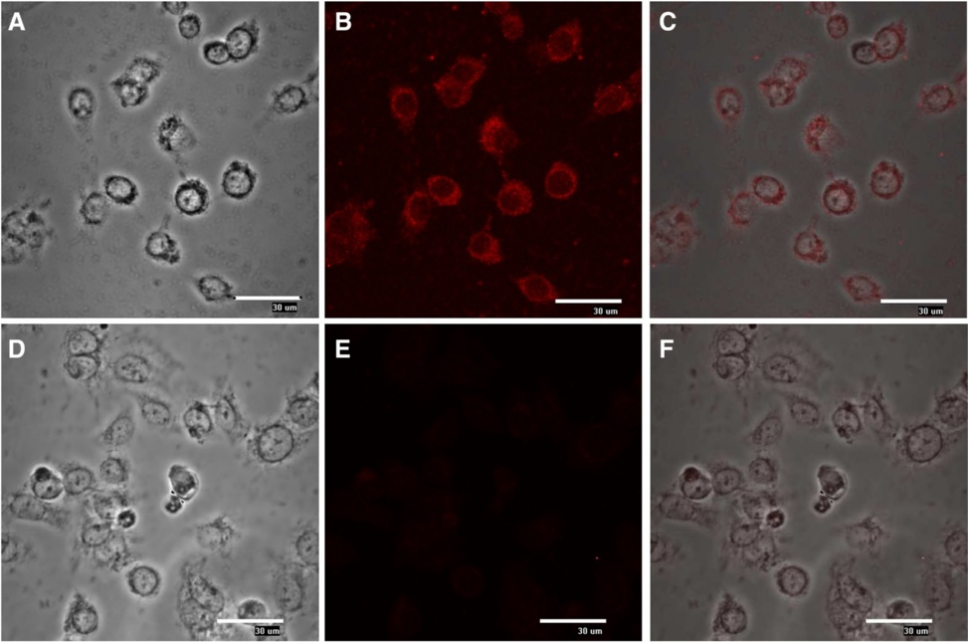
Permissivity of C33-A cells to DENV-2 infection. Cells were infected with dengue virus (MOI = 0.1) for 2 h, maintained for 24 h, washed, and fixed with methanol-acetone. The virus was detected using antibodies against the viral pr-M protein and a Texas Red secondary antibody and observed with fluorescence microscopy. **a**–**c** DENV-2-infected cells. **d**–**f** Mock-infected cells. **a**, **d** White light. **b**, **e** Fluorescence, (**c**, **f**) Merged images. Bars = 30 μm

To study the response of IFN-λ during DENV-2 infection, we selected the epithelial cell line C33-A. Because DENV infection has not been reported infecting C33-A cells, we performed initial assays and analyzed whether these cells are permissive to infection. After DENV-2 replication in C33-A cells, viral titers in the supernatants at 48 h post-infection were quantified by lytic plaque assays in BHK-21 cells, meaning that infectious viral progeny was released from C33-A cells. Immunofluorescence analysis using a specific antibody against DENV pr-M protein (Fig. 2) confirmed that C33-A cells are permissive to DENV-2 infection. Specific fluorescence was found in the cytoplasm of infected cells. With these data we propose the C33-A cell line as a novel epithelial model to analyze the relationship between DENV-2 infection and IFN-λ.

### DENV-2 infection and IFN-λ1 increase the expression of IL28R1 in C33-A cells

**Fig. 3:**
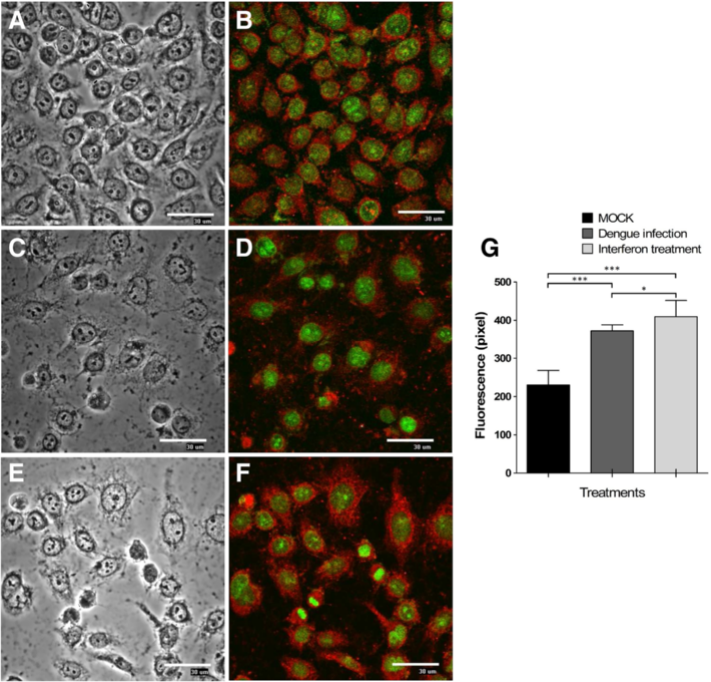
Membrane receptor expression IL28R in C33-A cells. The presence of IL28R1 was determined in C33-A cells: (**a**, **b**) Mock-infected. (**c**, **d**) DENV-2 infected (MOI = 0.1). **e**, **f** Treated with IFN-λ1 (35 ng/ml). Immunofluorescence was detected with anti-IL28R1 and CFL647-conjugated secondary antibody and observed by fluorescent microscopy (40X magnification). **g** Fluorescence intensities were determined by analysis of three different fields by randomly counting 50 cells using the image analysis software EZ-C1 v.2.3. *p < 0.05, ***p < 0.001. Nuclei were stained with the green fluorescent dye Sybr-14. Bars = 30 μm

To analyze the effect of DENV-2 infection on signal transduction initiated by IFN-III, the tissue-specific IL28R1 subunit of type-III interferon receptor was quantified on C33-A cells, by immunofluorescence. Figure 3b shows the presence of IL28R1 at significant levels on the cell membrane of non-infected cells (red signal). Expression of this subunit increased 76 % in the presence of IFN-λ1 (Fig. 3f) and 61 % during DENV-2 infection (Fig. 3d) with respect to untreated cells (quantification shown in Fig. 3g). Thus, DENV-2 and IFN-λ1 upregulate to a similar extent the expression of the IL28R1 subunit of the IFN-III receptor, suggesting that differential effects between these two stimuli may lie at other points in the IFN-λ signal transduction pathway.

### DENV-2 replication induces the expression of IFN-λ genes

**Fig. 4:**
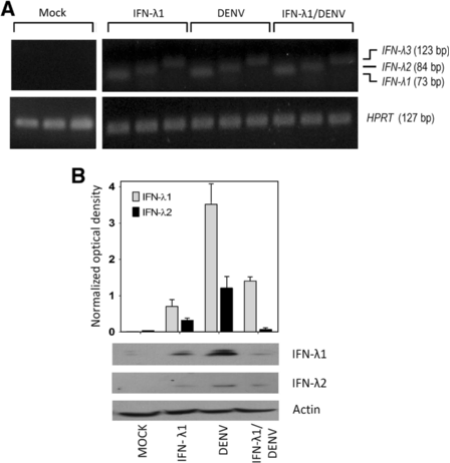
DENV-2 induces the expression of IFN-III in C33-A cells. C33-A cells were treated with IFN-λ1 (35 ng/ml) infected with DENV-2 (MOI = 0.1) or pre-treated with IFN-λ1 and subsequently infected with DENV-2. Forty-eight h post-infection, total cellular RNA was isolated and used in end-point RT-PCR for qualitative determination of the IFN-λ1, IFN-λ2, IFN-λ3 genes, using HPRT as an endogenous control gene. **a** Results of RT-PCR amplification of mock-infected and the three experimental conditions are shown. **b** Extracts of total protein from each condition were used for Western blotting to determine the presence of IFN-λ1 and IFN-λ2 proteins. Actin was the loading control. A semi quantitative analysis of detected bands normalized to actin was performed using ImageJ software (http://rsb.info.nih.gov/ij/)

To determine whether DENV-2 induces the expression of all subtypes of IFN-λs, four treatments were compared: Mock infection, IFN-λ1 treatment only (35 ng/ml), DENV-infection only (MOI = 0.1), and IFN-λ1 treatment followed by DENV infection. Mock-infected cells had no RT-PCR product for any of the IFN-λ isoforms (Fig. 4a). In contrast, IFN-λ1, IFN-λ2 and IFN-λ3 transcripts were demonstrated by end-point RT-PCR under the other three conditions studied (Fig. 4a).

To corroborate this result, total protein extracts from each condition were used for Western blot with specific antibodies against two subtypes of IFN-III (λ1 and λ2). Figure 4b shows that IFN-λ1 and IFN-λ2 are strongly induced by DENV-2 infection.

### IFN-λ1 and IFN-λ2 inhibit DENV-2 replication in a dose-dependent manner

**Fig. 5:**
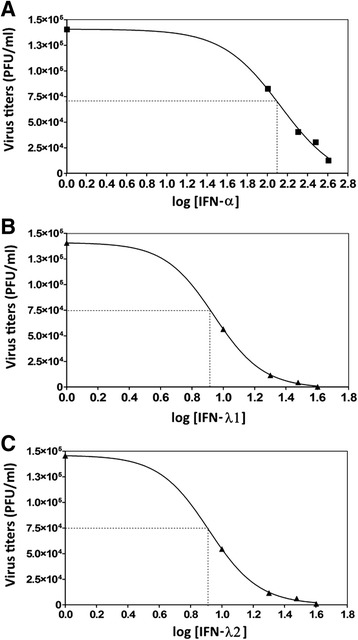
IFN-λ inhibits DENV-2 replication in a dose-dependent manner. C33-A cells were treated with increasing concentrations of IFN-α (**a**), IFN-λ1 (**b**) or IFN-λ2 (**c**) and then infected by DENV-2 (MOI = 0.1). Forty-eight hours post-infection, the viral titer was obtained by plaque assay. The doses used were 10–40 ng/mL for IFNλ1 or IFN-λ2 and 100–400 UI/ml for IFN-α. Plots show the percentage inhibition of the viral titer in each group compared to untreated cells. Dotted lines represent the IC50 values corresponding to IFN-λ1 = 8.705 ng/ml, IFN-λ2 = 8.24 ng/ml, IFN-α = 122–138 IU/ml. The points are representative of two independent experiments performed in triplicate. Experiments were fit to the Hill equation, using nonlinear regression to estimate the IC50 of IFN-λ1, IFN-λ2 or IFN-α (concentration of IFN that inhibited DENV-PFU production by 50 %, compared with parallel IFN free infected cultures). These IC50 values were considered for subsequent experimental procedures

To explore the effect of IFN-λ1 and IFN-λ2 on the DENV-2 infection, a replication inhibition curve was determined. C33-A cells were pretreated with increasing concentrations of IFN-λ1, IFN-λ2 (0, 10, 20, 30 and 40 ng/ml) or IFN-α (0, 100, 200, 300 and 400 IU/ml) prior to infection with DENV-2 (MOI = 0.1). Forty-eight hours after infection, the supernatants from each condition were quantified by lytic plaque-forming assays (Fig. 5).

Figure 5 shows that IFN-λ1 and IFN-λ2 inhibited DENV-2 replication in a dose-dependent manner, with relatively high inhibitory effect: IC50 = 8.705 ng/ml, IC90 = 17.78 ng/ml for IFN-λ1 (Fig. 5b) and IC50 = 8.24 ng/ml, IC90 = 16.2 ng/ml) for IFN-λ2 (Fig. 5c); whereas the IC50 for IFN-α was 122–138 IU/ml and IC90 = 380-398 IU/ml (Fig. 5a). These experiments showed that exogenous administration of IFN-λ prior to infection reduces the viral titer of DENV-2.

**Fig. 6:**
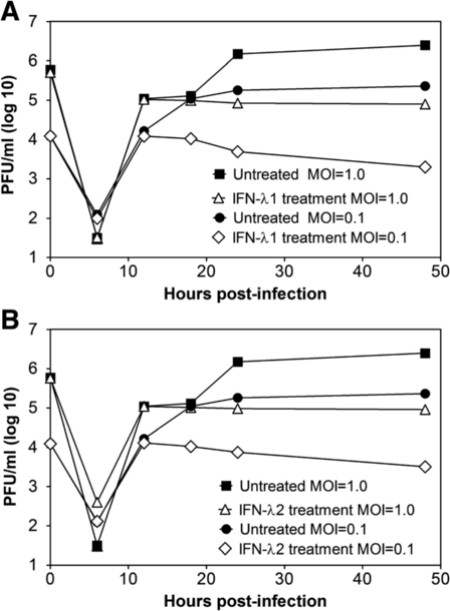
Kinetics of DENV-2 infection inhibition by IFN-λ. C33-A cells were untreated or treated with 10 ng/ml of (**a**) IFN-λ1 or (**b**) IFN-λ2 for 6 h prior to infection with DENV-2, which was performed at two different infectious doses (MOI = 0.1 and 1.0). The viral supernatant was titrated by plaque assays at 0, 6, 12, 18, 24 and 48 h post-infection. The points are representative of two independent experiments performed in triplicate

Subsequently, viral replication in the C33-A cells was determined on lytic plaque-forming assays at different time post-infection (0, 6, 12, 18, 24 and 48 h). Two infectious doses (MOI = 0.1 and 1.0) were assayed. Cells pretreated with 10 ng/ml of IFN-λ1 (Fig. 6a) or IFN-λ2 (Fig. 6b) for 6 h prior to infection were compared to cells that were infected but not treated with IFN. The plots show an equivalent effect of IFN-λ1 and IFN-λ2 pre-treatment on viral titers, which were reduced by 1.5–2 logs with respect to mock-treated cells.

### IFN-λ1 inhibits DENV-2 replication in cells lacking type I IFN

**Fig. 7:**
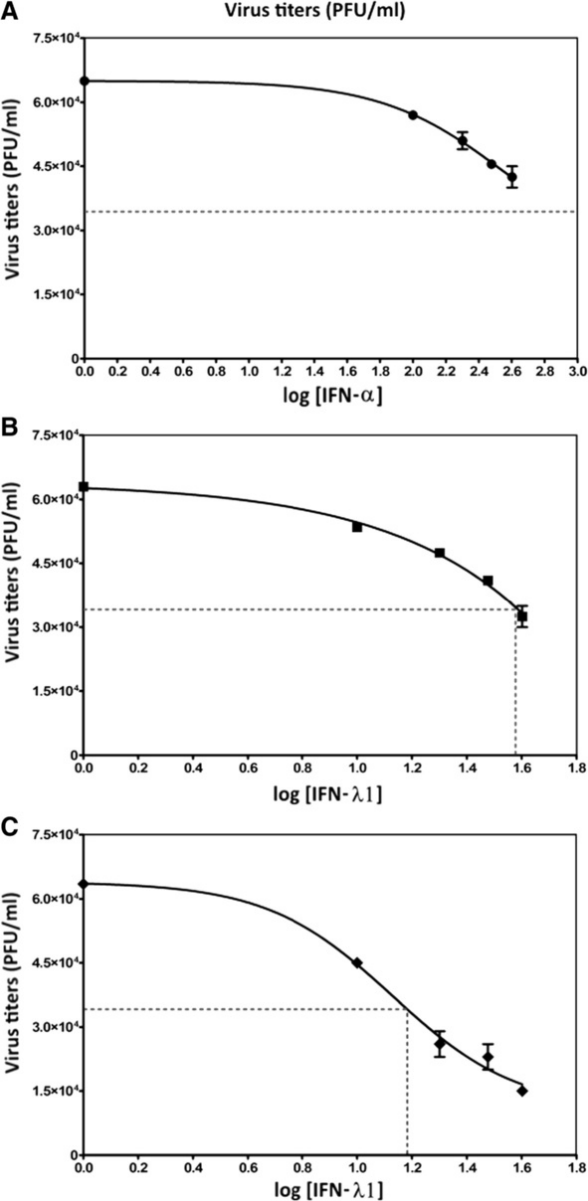
IFN-λ1 inhibits DENV-2 replication in cells lacking type I IFN. Vero cells were treated with increasing concentrations of IFN-α (**a**) or IFN-λ1 (**b**) or IFN-λ1 plus IFN-α (**c**), and then infected by DENV-2 (MOI = 0.1). The doses used were 10–40 ng/ml of IFN-λ1 or 100–400 UI/ml of IFN-α. Forty-eight hours post-infection, the viral titer was obtained by plaque assay. Plots show the percentage inhibition of the viral titer in each group compared to untreated cells. Dotted lines represent the IC_50_ values corresponding to IFN-λ1 = 37.15 ng/ml, IFN-λ1 in presence of IFN-α = 14.12 ng/ml, the value of IC50 for IFN-α was not calculated because 50 % inhibition was not reached under the conditions of experiment. Error bars represent standard deviations of means from two independent experiment *p < 0.01, **p < 0.001

To determine whether IFN-λ1 is enough to inhibit replication of DENV-2, we tested its ability to inhibit viral replication in the absence of INF-α. Inhibition experiments were similar to those described in Fig. 5, but they were performed on Vero cells that do not produce IFN-α, yet respond to it if supplied externally [21]. Vero cells were pre-incubated for 6 h with increasing concentrations of IFN-α, IFN-λ1 or IFN-λ1 plus IFN-α and then infected with DENV-2. By itself, IFN-λ1 treatment decreased the viral progeny 50 % at 37.15 ng/ml (IC50) (Fig. 7b). Treatment with IFN-λ1 plus IFN-α had a lower IC50 of 14.12 ng/ml calculated for IFN-λ1 (Fig. 7c); while for IFN-α treatment alone, IC50 couldn’t be calculated because the 50 % inhibition was not reached under the experimental conditions (Fig. 7a). This experiment suggests that IFN-λ1 is able to inhibit DENV-2 infection, but an effective response likely involves both types of interferons and a possible synergism between them.

### Pre-treatment with IFN-λ1 induces expression of ISGs that prevents viral replication

Expression kinetics of IFN-stimulated genes (ISGs) was analyzed in C33-A cells by quantitative real-time PCR (RT-qPCR) for the same experimental groups described in Fig. 4 (Mock infection, IFN-λ1 treatment, DENV-infection and IFN-λ1 followed by DENV infection). For this analysis, we selected the OAS1 and MX1 genes as indicative of interferon pathway functionality for the antiviral response, the IFN-λ1 and IFN-β genes, and two negative regulatory genes, SOCS1 and SOCS3.

**Fig. 8:**
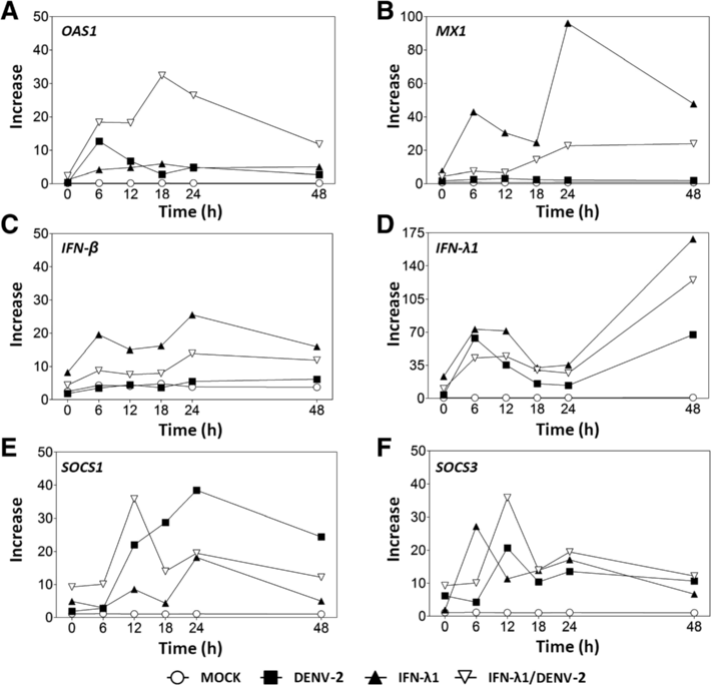
Transcription levels of IFN-stimulated genes during DENV-2 infection. Expression of OAS1 (**a**), MX1 (**b**), IFN-β (**c**), IFN-λ1 (**d**), SOCS1 (**e**), and SOCS3 genes (**f**) in cells infected with DENV-2 (MOI = 0.1) or treated with 10 ng/ml of IFN-λ1 or treated with IFN-λ1 before DENV-2 infection, were determined by RT-qPCR at 0, 6, 12, 18, 24, and 48 h post-infection or post-treatment. Results are presented as expression relative to untreated cells, which have a value of 1. mRNA levels were normalized to the HPRT gene. Data points represent the mean of three replicates and error bars indicate standard deviation

Figure 8a shows that stimulation of C33-A cells with IFN-λ1 slightly increases the transcription of OAS1 to similar levels between 6 and 48 h post-treatment (pt). DENV-2 infection significantly increases OAS1 mRNA but at earlier times, peaking at 6 h post-infection. However, the most striking effect was present with IFN-λ1 pre-treatment followed by DENV-2 infection, that showed a pronounced increase in OAS1 expression from 6 to 18 h post-infection, at which time it peaked and then underwent a slow steady decrease, remaining high at 24 and 48 h. This result suggests that IFN-λ1 decreases DENV-2 replication in part by viral mRNA degradation via RNase L. Moreover, stimulation of cells with IFN-λ1 dramatically increases MX1 gene transcription, suggesting that the efficient activation of IFN-III signal transduction is useful in controlling DENV-2 infection. IFN-λ1 treatment induced a bimodal MX1 increase with a first peak at 6 h post-infection and a second peak nearly 100-fold higher than basal at 24 h (Fig. 8b). Bimodality probably is due as a result of a first wave of activation by IFN-β and IFN-λ1 and subsequently, the increase at 24 h could be by IFN-λ2, which has demonstrated late induction, as well as by the late wave of endogenous interferons [22]. MX1 mRNA was detected at similar levels in infected and non-infected cells at all the studied times, suggesting that DENV induces an impairment in IFN signal transduction; however, this experiment cannot discriminate between IFN-I or IFN-III specific pathways. In cells pre-treated with IFN-λ1 and then infected, MX1 mRNA increased between 18 and 48 h post-infection, although not as much as OAS1.

To determine if DENV-2 has differential effects on the activation of IFN-I and IFN-III, mRNA levels of IFN-β (IFN-I) and IFN-λ1 (IFN-III) were quantified. Figure 8c shows that IFN-β mRNA is not increased at all in cells infected with DENV-2 at any of the studied times. IFN-λ1 stimulation increased IFN-β mRNA ~20–30 times between 6 and 24 h post-treatment. In contrast, IFN-λ1 pre-treatment followed by DENV-2 infection showed a <10-fold increase in IFN-β mRNA, remaining much lower than with IFN-λ1 stimulation alone. Therefore, it is believe that DENV-2 could affect the transcription of the IFN-β gene, probably by affecting IRFs.

In contrast, the three experimental conditions induced intense, bimodal stimulation of IFN-λ1 expression, with peaks at 6 and 48 h post treatment (Fig. 8d). IFN-λ1 treatment alone, increased IFN-λ1 mRNA 70-fold at 6 h and 170-fold at 48 h post-treatment; while DENV-2 and IFN-λ1 treatment followed by infection increased IFN-λ1 levels somewhat less (65-fold and 55-fold for DENV-2 and 40-fold and 120-fold for IFN-λ1/DENV-2, at 6 h and 48 h post-infection, respectively). The similar expression kinetics in Fig. 8b and c, in contrast to Fig. 8d, suggests that the inhibition of MX1 transcription in DENV-2 infection could be due primarily to lack of IFN-I.

Other important factors in the IFN pathway are the negative regulators SOCS1 and SOCS3, which increased from 12 h post infection (Fig. 8e and f). It is noteworthy that DENV-2 infection induces a significant SOCS1 increase that peaks at 24 h (40-fold), whereas in IFN-λ1 stimulation and subsequent infection this increase was impaired and reached only 20-fold at 24 h.

### Increase in SOCS1 mRNA coincides with a decrease in IFN-λ1 transcription

**Fig. 9:**
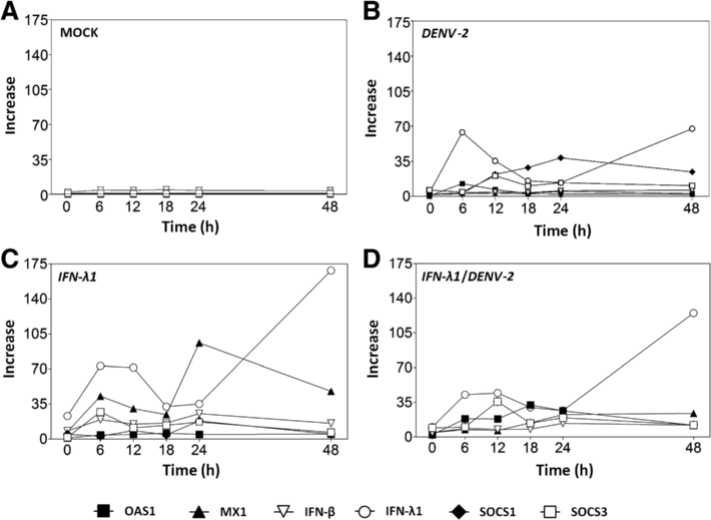
Increase in SOCS1 coincides with a fall in IFN-λ1 transcription. The same values used in the graphs of Fig. 8 were re-plotted and sorted according to experimental condition: (**a**) mock-infected cells, (**b**) infected with DENV-2 (MOI = 0.1), (**c**) treated with 10 ng/ml of IFN-λ1 or (**d**) treated with IFN-λ1 prior DENV-2 infection. Data points represent the mean of three replicates and error bars indicate standard deviation

In order to analyze other aspects of expression kinetics, the same data from Fig. 8 were re-plotted in Fig. 9 but grouped by experimental condition. Control cells (mock-infected) did not show significant activation of any gene tested, indicating that an external stimulus is required to activate their transcription in the evaluated conditions (Fig. 9a).

IFN-λ1 mRNA peaked at 6–12 h and 48 h post-treatment (Fig. 9b). DENV-2 infection stimulated the transcription of IFN-λ1, but the maximum value at 48 h post-treatment and was 65 % lower than in IFN-λ1 treated cells (open circles in Fig. 9b vs c).

For OAS1, the highest mRNA level was observed in cells treated with IFN-λ1/DENV-2 at 18 h post-infection (Fig. 9d). DENV-2 infection increased the IFN-λ1 transcription compared to non-infected cells treated with IFN-λ1. Conversely, the highest level of MX1 transcript was observed in the IFN-λ1-treated group at 6 h (40-fold) and 24 h (90-fold) (Fig. 9c). In addition, a steady increase in MX1 starting at 12 h post-infection and reaching 25-fold, was observed in the IFN-λ1/DENV-2 group (Fig. 9d). This increase was smaller than in cells stimulated only by IFN-λ1 (Fig. 9c) and it is of particular interest because the DENV-2-infected group did not show a significant increase in MX1 expression at any of the evaluated times.

The IFN negative regulators SOCS1 and SOCS3 showed maximum amounts at 6–12 h and 24 h post-stimulation with IFN-λ1. DENV2 infection also elicited an increment in their expression which was much stronger for SOCS1. Their activation coincided with that observed after DENV-2 infection but was even stronger. The IFN-λ1/DENV-2 experimental condition showed the highest expression of both genes at 12 h post-infection but it dropped to 40 % of the DENV-2-only expression level by 48 h post-infection. It was also evident that the peak of SOCS1 expression during DENV-2 infection, coincided with a drop in IFN-λ1 expression (Fig. 9b).

## Discussion

IFN-III is the most recently identified interferon group [10–12], and there is limited information about its expression and biological functions. The particular role of IFN-III during DENV infection is unexplored. Although most cells respond to IFN-I, certain cell types, especially epithelial cells, can respond to IFN-III due to the restriction in receptor IL28R1 expression [23, 24].

In the present work we demonstrated that dengue fever patients had ~1.8 times higher IFN-λ levels compared to healthy blood donors (*p* < 0.001). We are conducting further studies to answer whether IFN-λ plays a role in viral clearance or in the generation of specific clinical outcomes. For IFN-α, for instance, a recent study found higher levels in DF than DHF patients infected by DENV-1 or 2, that declined rapidly at day 3 after fever onset [25]. Such studies require the analysis of many patients, the determination of disease severity and the inclusion of patients infected with different DENV serotypes, among other variables.

Epithelial cells are IFN-III’s main target. An analysis of IL28R1 receptor expression in mouse cells showed that vaginal epithelium and keratinocytes but not fibroblasts respond to IFN-λ [23]. Several studies have been conducted in cell lines and primary cell cultures to study IFN-λ response [26]. In the present study we found that the C33-A cervical, epithelial cell line is permissive to DENV-2 infection and that this cell line both produces and responds to IFN-λ. IL28R1, which is essential to support the antiviral action of IFN-λ, is expressed in C33-A cells. IL28R1 expression is inducible under viral infections, concurrent with the expression of IFN-λ [16]. Accordingly, IL28R1 expression increased during DENV-2 infection in C33-A cells, which could potentiate the effect of IFN-λ signalling, leading to stimulation of gene transcription for the antiviral response and to inhibition of DENV-2 replication; as has been demonstrated for HCV, HIV and HBV infections [15, 27].

The present study demonstrated the ability of IFN-λ in the inhibition of DENV-2 replication. IFN-λ reduced the amount of infectious particles secreted into the supernatant of C33-A cultures, quantified by plaque assays. Treatment with IFN-λ or IFN-α decreased the viral titer. Dose-response experiments showed that the IC50 values for IFN-λ1 and IFN-λ2 were 8.24 ng/ml and 8.705 ng/ml, respectively (Fig. 5). In view of these IC50 values, we tested whether an IFN- λ concentration of 10 ng/ml, which produces optimal gene induction in other viral infections [28, 29], could induce the expression of antiviral response genes during dengue infection. In a study involving West Nile virus (WNV), another member of the *Flaviviridae* family, induction of IFN-λ and JAK/STAT pathway activation was demonstrated in infected cells. IFN-λ was able to prevent WNV infection of naïve cells, but failed to inhibit viral replication in cells once infection had been established, likely due to IFN-λ inhibition [30].

Furthermore, we report the IFN-λ1-mediated inhibition of DENV-2 replication in Vero cells, which are devoid of IFN-I, but have functional IFN-III responses. These data suggest that dengue virus and the IFN-λ treatment can active ISGs without type I IFNs. However, pre-treatment with IFN-λ1 before dengue infection was not able to inhibit viral replication to the same degree in Vero as in C33-A cells, which shows that dengue virus can block activated signalling pathways that induce both type I and III interferons. Since viral titers did not decrease markedly with the highest IFN-λ1 doses used, the data suggest that viral replication can be inhibited, albeit weakly, by endogenous and exogenous IFN-λ1 in complete absence of IFN-I. Furthermore, the reduction in viral progeny observed with IFN-λ1+ IFN-α, suggests that both interferons work synergistically. Importantly, the MX1 gene can be induced in Vero cells stimulated with recombinant type I interferon, thus demonstrating that although these cells don’t produce IFN-I they respond to it [21, 31, 32].

The antiviral effect shown by IFN-III could occur through the stimulation of antiviral genes as cells were first treated with IFN-λ1 and then infected. It is noteworthy that DENV-2 infection induced ISG transcripts but that these did not reach the levels observed when cells were stimulated with IFN-λ1. However, when cells were pre-treated with IFN-λ1 and then infected, ISG transcript levels were significantly higher and were maintained longer than in the DENV-2-infected group. SOCS1 and SOCS3 were induced after ISGs reached their highest peaks, which may denote the negative action exerted on the IFN-activated JAK/STAT pathway. However, the analyzed ISGs in the presence of IFN-λ1 stimulation remained at low levels but were sufficient to reach a second expression peak capable of inhibiting viral replication at late stages, which explains the decrease in viral titers observed between 18 and 48 h (Fig. 6).

The antiviral genes MX1 and OAS1 showed an early activation at 6 h and peaked at 18 and 48 h, respectively, in the IFN-λ1/DENV-2 group. OAS1 mRNA was higher when cells were pre-treated with IFN-λ1 and subsequently infected compared to cells that were stimulated only with IFN-λ1. This outcome was likely due to increased activation of the gene in the presence of infection plus gene activation mediated by interferon.

OAS1 has antiviral activity against DENV-2 infection [33]. The OAS/RNase L antiviral system is induced via IFN and plays a critical role in innate immunity, controlling the production of viral particles. RNase L possesses antiviral action, cleaving the viral RNA, inducing apoptosis in infected cells, and increasing the production of IFN-β through MDA5/RIG-I/IPS-I [34].

Higher activation of the MX1 gene occurs through stimulation of IFN-λ1 because it is a gene-specific interferon response, which indicates the proper functioning of the pathway in the cell line. In the case of the IFN-λ1/DENV-2 experiments, increased expression was most likely caused by pre-treatment with IFN-λ1 because the DENV-2-infected group showed no transcript increase for this gene. It is significant that although the transcript levels were as high as when cells were stimulated only with IFN-λ1, the observed levels may be sufficient to inhibit viral replication even with the activation of the SOCS genes, whose levels appear not be sufficient to completely inhibit the transcription of MX1 at later times.

Transcription levels of ISGs in different groups showed a clear relationship between treatment with IFN-λ and its role in inhibition of DENV-2 replication. It is possible to observe a process of evasion of the immune response, most likely mediated by viral infection, showing a decrease in the transcription of evaluated ISGs in DENV-2 infected cells compared with cells stimulated with IFN-λ and an increase in SOCS1 and SOCS3 genes.

However, the IFN-λ1 gene was activated initially and then in the last times evaluated (6 and 48 h post-infection), suggesting its escape from the suppressor activity of SOCS proteins. When cells were stimulated with IFN-λ1 and then infected, there was a slight and steady increase in the evaluated ISGs, which peaked at 12 or 18 and 48 h post-infection, suggesting that activity of the pathway is only partially affected by viral evasion. These findings suggest that IFN-I and IFN-III are not always concomitantly expressed and support the idea that there are differences in the regulatory mechanisms of their expression and in the group of genes that can be individually or jointly activated [35].

It is noteworthy that the highest peaks of the evaluated ISGs (12 and 18 h) corresponded to the time when the viral titer was reduced in culture supernatants; thus, greater inhibition of viral titer was achieved when ISGs reached their peaks (48 h). These findings suggest that inhibition of viral titer was caused by IFN-λ treatment and by the activation of genes in response to interferon, whose antiviral action in the cells inhibited the replication of DENV-2.

IFN-λ production increases after treatment with IFN-α prior to viral infection [36, 37]. Our results suggest that IFN-III enhances the expression of IFN-I as pre-treatment with IFN-λ1 increased IFN-β expression during infection with dengue virus. This leads us to propose that the crosstalk between interferon type I and III pathways may exist in some cells, like mononuclear or plasmacytoid dendritic cells, to produce and respond to both types of interferon [38, 39]. However, these are not necessarily redundant systems as shown in our experiments, wherein the expression of IFN-λ1 exists despite the evasion of the immune response mounted by DENV-2.

In the absence of antiviral drugs against DENV, this paper suggests that interferon lambda could be a valuable candidate to explore in clinical studies. In addition, the immunomodulatory properties reported for IFN-λ [13] can contribute to the regulation of the inflammatory process characteristic of dengue. Studies with IFN-α in Japanese encephalitis models have shown decreased inflammation [40], but treatment with IFN-α has side effects associated with the ubiquitous expression of its receptor [19, 20, 41]. In contrast, the narrow expression of type III interferon receptor could considerably diminish side effects of IFN-λ clinical use.

## Conclusions

This is the first report of increased levels of IFN-III in dengue fever patients *in vivo* and of the antiviral effect of IFN-III on DENV-2 infection *in vitro*; however, aspects of the activation of this pathway and its crosstalk with IFN-I remain unanswered.

The epithelial cell line C33-A is a useful model for *in vitro* mechanistic studies on DENV replication and its interaction with the innate response led by IFN-III. In infection studies, IFN-λ1 and IFN-λ2 inhibited DENV-2 replication in a dose-dependent manner. In Vero cells, which do not produce IFN-α, viral replication inhibition by exogenous IFN-λ1 was also observed, although at higher concentrations, suggesting that IFN-λ1 itself can inhibit the infection, however the potential to decrease the viral load increases with the presence of IFN-α. DENV-2 infection induces IFN-λ1 expression but not of IFN-β. Exogenous IFN-λ1 induced the transcription of several ISGs whose expression peaks corresponded to the time when viral titer was reduced in culture supernatants (12, 18, and 48 h post-infection). The present work opens the possibility for the study of other interactions and mechanisms of DENV infection with IFN-III in this cell model and presents an opportunity to consider this type of IFN as a feasible candidate for the inhibition of infection in dengue patients.

## Methods

### Cells

Human cervical carcinoma C33-A (ATCC: CRM-HTB-31), African green monkey kidney Vero (ATCC: CCL81, kindly donated by Dr. Judith González, Universidad Autónoma del Estado de Morelos, Mexico) and baby hamster kidney BHK-21 (C-13) (ATCC: CCL-10) cell lines were maintained in Dulbecco’s Modified Eagle’s Medium (DMEM) supplemented with 10 % fetal calf serum, 100 U/mL penicillin and 100 μg/mL streptomycin at 37 °C with 5 % CO_2_. *Aedes albopictus* C6/36 cells (kindly donated by Dr. Celso Ramos, Instituto Nacional de Salud Pública, Cuernavaca, Mexico) were maintained in Minimum Essential Medium (MEM) supplemented with 10 % fetal calf serum, 100 U/mL penicillin and 100 μg/mL at 27 °C.

### Virus

Dengue virus serotype 2 (Thailand/16681/1984) (DENV-2) used in this study was kindly provided by Dr. Alvaro Aguilar-Setien. DENV-2 was replicated in C6/36 cells and titrated by plaque assay in BHK-21 cells [42].

### Clinical samples

Serum samples from dengue patients collected from June to September 2013, were analyzed to determine the IFN-λ. All serum samples were maintained at -70 °C, thawed onto ice and immediately subjected to ELISA. Patients were recruited in the Regional Hospital at Mepetec, Puebla, Mexico. All patients had signs of dengue fever and DENV infection was confirmed by IgM antibodies and viral NS1 antigen detection. The samples from included patients were obtained between days 3 and 5 after fever onset. Sampling and patient information management were performed following ethical regulations approved by Local Research and Health Ethics Committee #2101 of Mexican Institute for Social Security, IMSS (Protocol: R-2011-2103-24) and in accordance with the Declaration of Helsinki. Written informed consent was obtained from each participant. Controls were serum samples of healthy blood donors submitted to conventional studies at the hospital blood bank and were used to compare IFN-λ levels.

### Infection assays

C33-A or Vero cells were seeded onto 12- or 24-well plates and infected with different virus doses [quantified by multiplicity of infection (MOI)]. Viral inocula were incubated for 1 h and then discarded. Cells were washed twice to eliminate non-adsorbed virus. Fresh medium (SFB 2.5 %) was added to the cells and then incubated for hours or days depending on the specific assay.

### Cell stimulation

Cells were treated prior to infection for 6 h with 100–500 UI/ml of IFN-α (Probiomed, Mexico city, Mexico.), 10–50 ng/ml of IFN-λ1 (PeproTech, Rocky Hill, NJ, USA) or 10–50 ng/ml IFN-λ2 (PeproTech). A poly(I:C) (InvivoGen, San Diego, CA, USA) control treatment was performed on cells using DMEM without SFB and 100 μg/ml of poly(I:C).

### Western blot

Infected cells or mock-infected cells, previously treated or mock-treated with IFNs for 6 h, were analyzed in order to determine the induction of IFN-λ1 and IFN-λ2. Total extracts of cells were obtained 48 h post-infection and subjected to SDS-PAGE using a 15 % resolving gel. Proteins in gel were transferred to PVDF membranes and detected by anti-IFN-λ1 (Santa Cruz Biotechnology, Santa Cruz, CA, USA, sc-67642), anti-IFN-λ2 (Abcam, Cambridge, MA, USA, ab109820) and anti-β-actin specific antibodies (Abcam, mAbcam8226). Primary antibodies were revealed with a goat anti-rabbit IgG-HRP (Santa Cruz Biotechnology, sc-2004), rabbit anti-goat IgG-HRP (Abcam, ab6741) or goat anti-mouse IgG-HRP (ab97023) and the Immobilon Western chemiluminescence system (Millipore-Merck, Darmstadt, Germany).

### Fluorescence microscopy

C33-A cells were seeded onto glass chamber slides (Lab-Tek, Nalgene Nunc, Rochester, NY, USA) and infected as mentioned above. Infected or non-infected controls were washed with phosphate-buffered saline (PBS) and fixed in ethanol/acetone 50 % v/v for 10 min at room temperature. Cells were permeabilized with 0.2 % Triton-X100 for 2 min in order to detect the viral pr-M protein inside infected cells and incubated in blocking solution (PBS with 10 % FBS and 0.2 % Tween 20). Cells were labelled with anti-dengue virus pr-M antibody (Abcam, ab41473) or anti-IL28R (Santa Cruz Biotechnology, sc-66541), washed and incubated with rabbit anti-mouse IgG-Texas red (Abcam, ab6726) or bovine anti-goat IgG-CFL-647 (Santa Cruz Biotechnology, sc-362284) for 1 h at room temperature. SYBR-14 dye (Molecular Probes, Eugene, OR, USA) was used to stain the nuclei. The stained cells were visualized by laser confocal microscopy system E600-C1 using the image analysis software EZ-C1 (Nikon).

### Real time PCR

Total RNA of DENV-2 infected and/or stimulated cells was extracted using Trizol (Invitrogen, Carlsbad, CA, USA) according to the manufacturer’s instructions, treated with DNase I (Thermo Scientific, Waltham, MA, USA) and quantified at 260/280 nm. Reverse transcription was performed using RevertAid H Minus Reverse Transcriptase kit (Thermo Scientific) and random hexamers. The reaction program was 25 °C for 10 min, 42 °C for 60 min and 70 °C for 10 min. The obtained cDNA was used to amplify the IFN-β, OAS1 and MX1 genes using the Taqman Universal PCR Master Mix, no AmpErase UNG kit (Applied Biosystems, Foster City, CA, USA). IFN-λ1, SOCS1 and SOCS3 genes were amplified using Maxima SYBR Green/ROX qPCR Master Mix (Thermo Scientific). The reaction mixture contained 5 μl 2X Master Mix, 200 nM of each sense and antisense primer, 200 nM of Taqman probe and 35–50 ng RNA in a final volume of 10 μl. Forty PCR cycles were performed at 50 °C for 2 min, 94 °C for 10 min, 95 °C for 15 s, 60 °C for 30 s and 72 °C for 30 s in a StepOne Real-Time PCR System (Applied Biosystems). Specific primers for IFN-λ1, IFN-β, OAS1 and MX1 genes were designed using ProbeFinder Assay Design Software (Roche, Basel, Switzerland, www.universalprobelibrary.com) in order to use the Taqman probes of Universal ProbeLibrary Set, Human (Roche). In all cases, HPRT1 (Hypoxanthine Guanine Phosphoribosyl Transferase) gene was used as an internal control to determine the relative expression of each gene. All primers were purchased from Integrated DNA Technologies (Coralville, IA, USA). Obtained Ct values were analyzed by the 2 − ΔΔCt method, and the expression levels were reported as proportions with respect to controls. Sequences of primers and probes are shown in Table 1.

**Table 1:** Primers used in the present study

Gene name and accession number in GenBank	Sequence (5′–3′)	UPL sequence (UPL#)^a^	Amplicon bp	Alignment^b^
MX1	TCCAGCCACCATTCCAAG	TTCTCCTG (2)	60	868–885
NM_001144925.2	CAACAAGTTAAATGGTATCACAGAGC			927–902
OAS1	GGTGGAGTTCGATGTGCTG	CCAGGGCA (37)	64	538–556
XM_006719434.1	AGGTTTATAGCCGCCAGTCA			601–582
IFN-β	CTTTGCTATTTTCAGACAAGATTCA	CTGGCTGG (20)	69	336–360
NM_002176.2	GCCAGGAGGTTCTCAAGAAT			404–389
IFN-λ1	TCCTAGACCAGCCCCTTCA	^c^	73	435–453
NM_172140.1	GTGGGCTGAGGCTGGATA			507–490
IFN-λ2	CCTGGTGGACGTCTTGGA	^c^	84	406–423
NM_172138.1	GTGGGCTGAGGCTGGATA			489–472
IFN-λ3	AGGGCCAAAGATGCCTTAG	^c^	123	167–185
NM_172139.2	CAGCTCAGCCTCCAAAGC			298–281
HPRT1	–	–	127	–
NM_000194.2^d^				

^a^UPL: Universal Probe Library, Roche Life Science. Number of the probe is in parentheses

^b^Position of primers in the corresponding gene sequences

^c^SybrGreen dye was used

^d^Specific gene positions and sequences of Applied Biosystems primers and probes are not disclosed

### ELISA

Levels of IFN- λ in the serum of patients were measured using enzyme-linked immunosorbent assay (ELISA) kit specific for IFN-λ1 and λ3 (DY1598B, R&D systems, Inc. Minneapolis, USA). The ELISA was performed according to the manufacturer’s instructions.

### Statistical analysis

Quantitative analyses were performed in triplicate in at least two different assays. Two-way ANOVA and Bonferroni test were performed using GraphPad Prism 5 (GraphPad Software, La Jolla, CA, USA). Differences with *p* < 0.05 were considered statistically significant.
